# Physiological responses to psychological stress: importance of adiposity in men aged 50–70 years

**DOI:** 10.1530/EC-14-0042

**Published:** 2014-05-26

**Authors:** S U Jayasinghe, S J Torres, C A Nowson, A J Tilbrook, A I Turner

**Affiliations:** Centre for Physical Activity and Nutrition Research School of Exercise and Nutrition Sciences, Deakin University, Burwood Melbourne, Victoria, 3125 Australia; 1 Livestock and Farming Systems South Australian Research and Development Institute, University of Adelaide, Roseworthy Adelaide, South Australia, 5371 Australia

**Keywords:** stress, sympatho-adrenal medullary system, hypothalamo–pituitary–adrenal axis, obesity, adiposity, men's health, cortisol

## Abstract

We tested the hypothesis that overweight/obese men aged 50–70 years will have a greater salivary cortisol, salivary alpha amylase and heart rate (HR) responses to psychological stress compared with age matched lean men. Lean (BMI=20–25 kg/m^2^; *n*=19) and overweight/obese (BMI=27–35 kg/m^2^; *n*=17) men (50–70 years) were subjected to a well-characterised psychological stress (Trier Social Stress Test, TSST) at 1500 h. Concentrations of cortisol and alpha amylase were measured in saliva samples collected every 7–15 min from 1400 to 1700 h. HR was recorded using electrocardiogram. Body weight, BMI, percentage body fat, resting systolic and diastolic blood pressure and mean arterial pressure were significantly higher (*P*<0.05) in overweight/obese men compared with lean men. Both groups responded to the TSST with a substantial elevation in salivary cortisol (372%), salivary alpha amylase (123%) and HR (22%). These responses did not differ significantly between the groups (time×treatment interaction for salivary cortisol, salivary alpha amylase and HR; *P*=0.187, *P*=0.288, *P*=0.550, respectively). There were no significant differences between the groups for pretreatment values, peak height, difference between pretreatment values and peak height (reactivity) or area under the curve for salivary cortisol, salivary alpha amylase or HR (*P*>0.05 for all). The results showed that, for men with a moderate level of overweight/obesity who were otherwise healthy, the response of salivary cortisol, salivary alpha amylase and HR to acute psychological stress was not impaired.

## Introduction

The ability of stress to impair physiological processes such as growth, reproduction and immune competence and its association with diseases such as cardiovascular disease, type 2 diabetes and anxiety and depression are well known (1). While acute responses to stress are generally considered effective in dealing with immediate threats, prolonged activation of stress processes could have significant adverse consequences for individuals (2). However, frequent exposure to acute activation of the stress pathways may also be detrimental. People with elevated responses to acute stress are more likely to have psychological disorders such as anxiety and depression (3, 4). They are also more likely to develop hypertension and have increased risk of developing cardiovascular disease (5, 6).

During stress, activation of the sympatho-adrenal medullary (SAM) system results in the release of noradrenaline from sympathetic nerve terminals and adrenaline and noradrenaline from the adrenal medulla, which result in a range of rapid physiological and behavioural responses such as increases in heart rate (HR) and blood pressure and heightened vigilance (7). Research also shows that activation of the SAM system increases the secretion of alpha amylase by the salivary glands (8, 9, 10, 11). Simple sampling procedures of saliva make salivary alpha amylase a useful non-invasive measure of the SAM system activity (12). Further advantages of salivary alpha amylase include its concentration being independent of the salivary flow rate (11) and that its response to Trier Social Stress Test (TSST) is predictive of the plasma catecholamine response to TSST (13).

Activation of the hypothalamo–pituitary–adrenal (HPA) axis by stress results in the sequential secretion of corticotrophin-releasing hormone (CRH) and arginine vasopressin (AVP) from the hypothalamus, adrenocorticotrophic hormone (ACTH) from the anterior pituitary gland and glucocorticoids such as cortisol from the adrenal cortex (14, 15). Secretion of cortisol results in the mobilisation of energy stores, which will help in responding to the emergency situation (14). Changes in the above parameters are used as markers of the activity of these stress pathways.

The magnitude of the response to psychological stress can be influenced by the physiological status of an individual (2). Obesity (a state of elevated adiposity) has reached epidemic proportions in the Western world (16). As such, an understanding of the influence of obesity on the responsiveness of the stress systems to stress is essential. Most of the research done in this area has been in women of different age groups (17, 18, 19). Given that there are gender differences in stress pathway responsiveness to stress (20, 21), findings in females cannot be extrapolated to men. Therefore a paucity of evidence exists in this area for men. Previous research in obesity and stress has reported both increased (22, 23), decreased (24, 25) as well as comparable (26) stress pathway activation in individuals with increased levels of adiposity compared with lean individuals in response to psychological stress. The inconclusive results could be due to differences in the stressor utilised, time of day, an inadequate baseline resting period and low frequency of sampling. Furthermore, very little research has considered the SAM system and the HPA axis together.

The aims of this study were to determine whether lean and overweight/obese men differ in their cortisol and salivary alpha amylase and HR responses to the TSST. It was hypothesised that overweight/obese men aged 50–70 years will have a greater salivary cortisol, salivary alpha amylase and HR response to the TSST compared with age matched lean men.

## Subjects and methods

### Participants

Lean (BMI range 20–25 kg/m^2^) and overweight/obese (BMI range 27–35 kg/m^2^) men aged 50–70 years were recruited using newspaper and online advertisements, fliers in community centres and medical clinics, mail outs to participants of previous studies and by fliers dropped in mail boxes. During recruitment, our originally intended BMI range for obese individuals of 30–35 kg/m^2 ^was relaxed to 27–35 kg/m^2^ (overweight/obese) to ensure an adequate sample size in this group. Exclusion criteria were prior diagnosis with Cushing's syndrome, any stress or anxiety disorder, depression, any diseases of the adrenal gland, type 2 diabetes, heart disease (including use of a pacemaker), high cholesterol, stroke or cancer. Information about these medical conditions was obtained by self-report from the participants via a telephone interview. At face-to-face screening, men were excluded if their BMI fell out of the required ranges or if their resting blood pressure exceeded 160 mmHg for systolic blood pressure or 90 mmHg for diastolic blood pressure.

Every participant provided written informed consent before participation in the study. All procedures were approved by the Human Research Ethics Committee of Deakin University (Project code: EC00213) and conformed to the guidelines of the National Health and Medical Research Council's National Statement on Ethical Conduct in Human Research (2007).

### Experimental procedure

**Figure 1:**
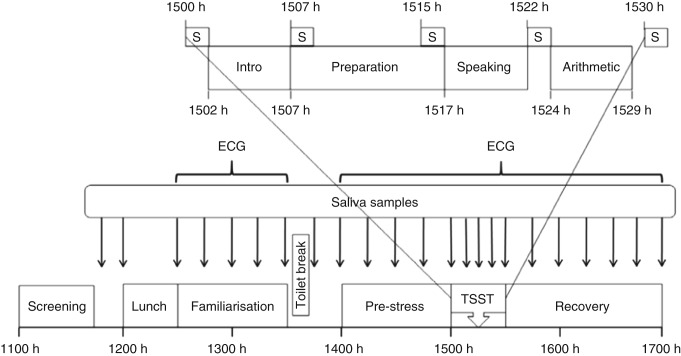
Schematic representation of the stress testing day. ECG, electrocardiogram; TSST, Trier Social Stress Test; S and black arrows, saliva samples.

During the 12 h before participation in the study, participants were asked to abstain from smoking, ingesting any caffeine-containing beverages (e.g. tea, coffee and cola), liquorice, alcohol or drugs (except for any regular medications) and from strenuous physical activity. A schematic representation of the experimental day is presented in Fig 1. Briefly, participants arrived at the research laboratory at 1100 h. Their eligibility was confirmed through the measurement of anthropometric variables and blood pressure. Eligible participants were then given a standardised lunch at 1200 h where they were allowed to make their own meal using bread, margarine, processed meat (ham or chicken), tomato, cucumber, cheese, nuts, fruit bars and a fruit drink (juice box). Records of foods consumed were kept. Water was available *ad libitum*. A TSST was imposed from 1500 to 1530 h. Saliva samples were collected every 15 min from 1145 until 1700 h apart from 1215 h (lunch) where no sample was collected and between 1500 and 1530 h where samples were collected more frequently (1500, 1507, 1515, 1522 and 1530 h). Electrocardiogram (ECG) was measured from 1230 to 1700 h, with the exception of the 10 min break to use the bathroom at 1330 h. Only data from the period 1400–1700 h are considered in this paper.

### Anthropometric measures

Participant's height was measured using an electronic stadiometer (Measurement Concepts, North Bend, Australia). Body weight was measured using a standard scale (TANITA, Wedderburn, Melbourne, VIC, Australia). BMI was calculated using the equation BMI (kg)/height (m)^2^. Percentage body fat and percentage lean mass were measured using bioelectrical impedance (TANITA, Wedderburn). Waist circumference was measured at the midpoint between the last rib and the anterior superior iliac spine, using a tape measure, and hip circumference was measured at the widest point of the gluteal area (27). Waist-to-hip ratio was calculated by dividing waist circumference by hip circumference. Resting systolic and diastolic blood pressures and mean arterial pressure were calculated as the average of the last three of four measurements taken at 2 min intervals using an automatic sphygmomanometer (Criticare Systems, Inc., Waukesha, WI, USA).

### Saliva sampling

Saliva samples were collected using Salivette sampling tubes (Sarstedt, Ingle Farm, SA, Australia) consisting of a centrifugation tube and a cotton swab. The participants were asked to hold the cotton swab in their mouth for 2 min. They were instructed to keep the cotton swab under their tongue during the first 30–45 s and move it around the oral cavity for the remainder of the duration without chewing it or holding it between their teeth. The samples were stored on ice after collection. After the testing period, samples were centrifuged at 639 ***g*** for 5 min at 4 °C. Resultant saliva was stored at −80 °C until assayed.

### Heart rate

A three lead ECG (ADinstruments, Castle Hill, NSW, Australia) was used to measure HR. One electrode was placed on each of the participants' wrists and another just above the antecubital fossa of the elbow. A power spectral analysis of the ECG data was conducted using Lab Chart Pro software (ADinstruments) to calculate HR. For data presentation and analysis, HR was calculated as the mean of data for 5 min blocks, commencing from 1 min before to 4 min after the commencement of each saliva sampling period.

### Trier Social Stress Test

The TSST is a well-characterised psychosocial stress protocol (28). Briefly, after a 1500 h sample collection, the test commenced with an introduction during which participants were introduced to the panel and were given instructions about the speaking task to follow (Fig. 1, inset). This was followed by a 10 min preparation phase with saliva samples collected at the beginning (1507 h) and end (1515 h) of the preparation phase. Five minutes of public speaking followed after which another saliva sample was collected (1522 h). The participants were given instructions regarding the mental arithmetic task at this point and 5 min of mental arithmetic followed. Another saliva sample was collected at the end of the mental arithmetic task (1530 h). Minor differences from the original protocol were that saliva samples were collected during the TSST (Fig. 1) including between the speaking and mental arithmetic tasks (not collected in the original protocol) and that there were two members of the interview panel (three members in the original protocol). The purpose of the saliva samples collected during the TSST (1507, 1515 and 1522 h, Fig. 1) was to capture any rapid changes in SAM system activity during the stressor.

### Saliva cortisol assays

Saliva concentrations of cortisol were measured using an enzyme immunoassay (Diagnostic Systems Laboratories, Webster, TX, USA). Thirty-one assays were conducted with a mean sensitivity of 0.035 μg/dl. The intra-assay coefficient of variation was 6.9% at 0.25 μg/dl and 8.2% at 2.0 μg/dl. The inter-assay coefficient of variation was 9.4% at 0.28 μg/dl and 7.7% at 1.8 μg/dl.

### Salivary alpha amylase assays

Saliva concentrations of alpha amylase were measured using a kinetic assay kit (Salimetrics, Carlsbad, CA, USA). Thirty-six assays were conducted with a mean sensitivity of 0.4 U/ml. The intra-assay coefficient of variation was 7.4% at 156.3±4.1 U/ml. The inter-assay coefficient of variation was 7.4% at 20.7 U/ml and 7.0% at 257.3 U/ml.

### Statistical analyses

#### Preliminary analysis

Pretreatment cortisol was defined as the average of the five concentrations from 1400 to 1500 h (1400, 1415, 1430, 1445 and 1500 h). Pretreatment salivary alpha amylase was defined as the average of the three concentrations from 1430 to 1500 h (1430, 1445 and 1500 h). Pretreatment HR was defined as the average of the four readings from 1400 to 1445 h (1400, 1415, 1430 and 1445 h). Peak height for all parameters was defined as the highest value that was obtained for each individual after the commencement of the stress. Reactivity was calculated by subtracting the pretreatment value from the peak height for all parameters. Area under the curve (AUC) (with respect to increase) was calculated for each parameter using all values from 1500 to 1700 h after the subtraction of the pretreatment value from each data point. Areas under the curve were calculated using the trapezoid rule utilising Sigmaplot graphing software (Systat Software, Inc., San Jose, CA, USA).

Recovery time for all parameters was defined as the time difference from the commencement of the stressor (1500 h) to the point at which the relevant parameter returned to within two s.d. of its pretreatment value. We used this as our definition for recovery, because 95% of a normally distributed set of data lie within two s.d. of the mean and because 5% error (*P*=0.05) is the generally accepted cut-off level for statistical significance. This indicates that once the value has returned to within two s.d. of the pretreatment level, it has returned to the pretreatment levels. For recovery time only, those who did not exceed two standard deviations of the pre-treatment value between 1500–1700 h (*n*=1 lean and *n*=2 overweight/obese for cortisol, *n*=2 lean and *n*=5 overweight/obese for salivary alpha amylase, *n*=0 lean and *n*=0 overweight/obese for heart rate) were excluded from the analysis. For those who did exceed two standard deviations but did not return to within two standard deviations by 1700 h (*n*=8 lean and *n*=6 overweight/obese for cortisol, *n*=4 lean and *n*=1 overweight/obese for salivary alpha amylase, *n*=0 lean and *n*=1 overweight/obese for heart rate), 120 minutes was used as the recovery time in the analyses.

#### Analysis

Data were analysed using the Statistical Package for the Social Sciences software version 20.0 for windows (SPSS, Inc.). Kolmogorov–Smirnov and Shapiro–Wilk tests were conducted to test for normality. Tests for homogeneity of variance were conducted using Levene's test of equality of error variances. Descriptive characteristics were compared between groups using univariate ANOVA. Salivary cortisol, salivary alpha amylase and HR were compared within and between groups using repeated measures ANOVA. The within subjects factor was time and the between subjects factor was treatment. Derived salivary cortisol, salivary alpha amylase and HR parameters (pre-treatment, peak height, reactivity and AUC) were compared between groups using univariate ANOVA. Pearson's correlation was used to test for relationships between pre-treatment, peak height, reactivity and AUC and measures of adiposity (BMI, percentage body fat, waist circumference, waist-to-hip ratio). *P*<0.05 was considered statistically significant.

We estimated that 32 participants in total (16 lean and 16 overweight/obese) were needed to find a difference between groups in salivary cortisol of the same magnitude as that found by Klaperski *et al*. (29) with a significance level of 0.05 and a power of 90%.

## Results

### Participants

Data were collected from 24 lean and 22 overweight/obese men who were eligible for the study. Two lean and five overweight/obese men were subsequently excluded from the analyses due to insufficient saliva volume to undertake the assays for cortisol and alpha amylase. Initial analysis revealed that the lean men were significantly older (64.2±1.1 years) than the overweight/obese men (61.0±1.1 years) (*P*=0.054). The results of the three oldest lean men were excluded from the analyses to remove the significant effect of age. Consequently, the results from 19 lean and 17 overweight/obese men (seven overweight and ten obese) were included in the analyses for cortisol and salivary alpha amylase. The individuals who were excluded did not differ significantly from the final cohort in any of the baseline characteristics. Including these three men in the final analyses did not change any of the outcomes. Due to technical problems with ECG recordings, data were not available for two further lean and two further overweight/obese men leaving 17 lean and 15 overweight/obese men (six overweight and nine obese) for HR analysis. The individuals who were excluded from the final analysis due to technical difficulties in ECG recording did not differ significantly from the final cohort in any of the baseline characteristics.

### Participants' characteristics

Overweight/obese men had a significantly (*P*<0.001) greater body weight and BMI compared with lean men (Table 1). On average, overweight/obese men had 7.9% more body fat compared with lean men (*P*<0.001). Overweight/obese individuals averaged a 19% larger waist circumference (*P*<0.001), a 10% larger hip circumference (*P*<0.001) and a 10% larger waist-to-hip ratio (*P*<0.001) compared with lean men. Resting systolic and diastolic blood pressure and mean arterial pressure were also significantly (*P*<0.05) higher in overweight/obese men compared with lean men (Table 1). Nevertheless, age, height and resting HR did not differ significantly between the groups (Table 1). At the lunchtime meal, the consumption of total energy, protein, carbohydrate, fat and sodium was similar between the groups (Table 1).

### Cortisol

**Figure 2:**
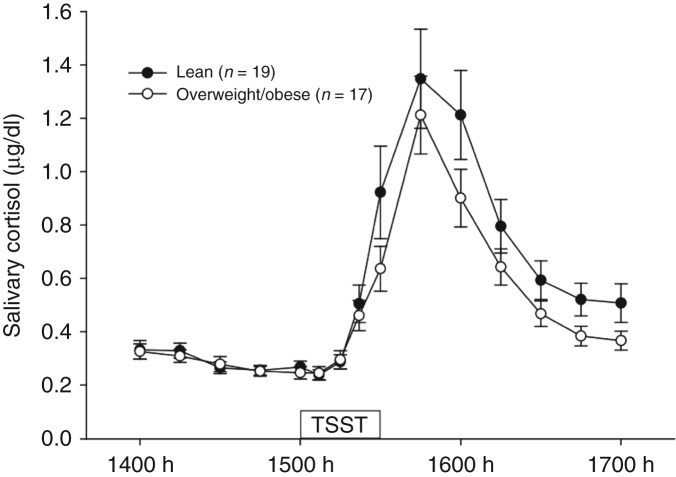
Mean (±s.e.m.) concentrations of salivary cortisol (μg/dl) in lean and overweight/obese men from 1400 to 1700 h (time effect *P*<0.001; time×treatment interaction *P*=0.187; treatment effect *P*=0.210). TSST, Trier Social Stress Test.

Saliva concentrations of cortisol in lean and overweight/obese men are shown in Fig. 2 and Table 2. Repeated measures ANOVA revealed that there was a significant effect of time (*P*<0.001; Fig. 2). Overall (both groups combined), the peak height of cortisol concentrations (1.37±0.19 μg/dl) was significantly higher than pretreatment concentrations (0.29±0.02 μg/dl) (*P*<0.001). Overall, there was a 372% increase in cortisol concentrations from pretreatment concentrations to the peak of the response (both groups combined).

Saliva concentrations of cortisol in response to the TSST did not differ significantly between lean and overweight/obese men (time×treatment, *P*=0.187; Fig. 1) and accordingly, there were no significant differences between the groups in peak height, cortisol reactivity or AUC for the cortisol response (Table 2). The mean time to recovery did not differ significantly between the groups (Table 2). There was no significant overall difference between the groups (between subjects effect; *P*=0.210).

There were no significant associations (data not shown) between measures of cortisol (pre-treatment cortisol, peak height, cortisol reactivity, AUC) and measures of adiposity (BMI, percentage body fat, waist circumference and waist-to-hip ratio).

### Saliva concentrations of alpha amylase

**Figure 3:**
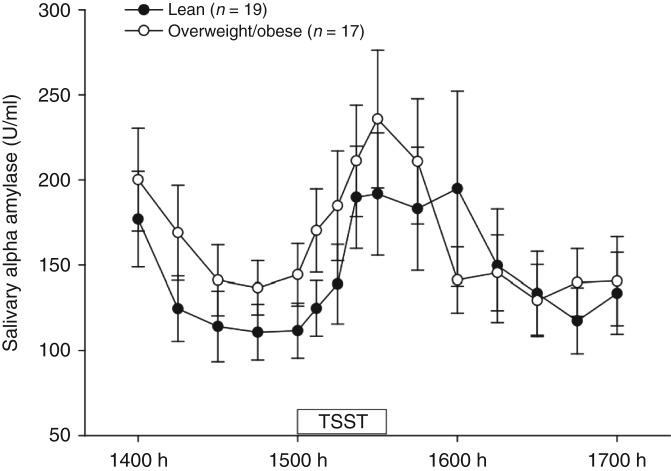
Mean (±s.e.m.) concentrations of salivary alpha amylase (U/ml) in lean and overweight/obese men from 1400 to 1700 h (time effect *P*<0.001; time×treatment interaction *P*=0.288; treatment effect *P*=0.322). TSST, Trier Social Stress Test.

Saliva concentrations of alpha amylase in lean and overweight/obese men are shown in Fig. 3 and Table 3. There was a significant time effect (*P*<0.001; Fig. 3). Overall (both groups combined), the peak height of salivary alpha amylase concentrations (281.5±48.35 U/ml) was significantly higher than pretreatment concentrations (126.5±16.3 U/ml) (*P*<0.001). Overall, there was a 123% increase in salivary alpha amylase from pretreatment concentrations to the peak height of the salivary alpha amylase response (both groups combined).

Salivary alpha amylase concentrations in response to the TSST did not differ significantly between lean and overweight/obese men (time×treatment, *P*=0.288; Fig. 3) and accordingly, there were no significant differences between the groups in peak height, reactivity or AUC of the salivary alpha amylase response (Table 3). The mean time to recovery did not differ significantly between the groups (Table 3). There was no significant treatment effect, indicating that there was no significant overall difference between the groups (*P*=0.332).

There were no significant associations (data not shown) between measures of salivary alpha amylase (pretreatment salivary alpha amylase, peak height, salivary alpha amylase reactivity, AUC) and measures of adiposity (BMI, percentage body fat, waist circumference and waist-to-hip ratio).

### Heart rate

**Figure 4:**
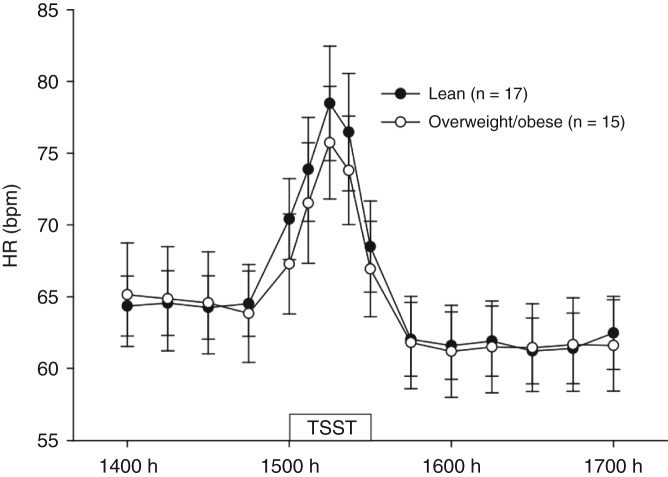
Mean (±s.e.m.) heart rate (bpm) in lean and overweight/obese men from 1400 to 1700 h (time effect *P*<0.001; time×treatment interaction *P*=0.550; treatment effect *P*=0.838). TSST, Trier Social Stress Test.

HR data in lean and overweight/obese men are shown in Fig. 4 and Table 4. There was a significant effect of time (*P*<0.001; Fig. 4). Overall (both groups combined), peak height of HR (78.6±4.1 bpm) was significantly higher than pretreatment values (64.5±3.9 bpm) (*P*<0.001). Overall, there was a 22% increase in HR from pretreatment values to the peak height of the HR response (both groups combined).

HR response to the TSST did not differ significantly between lean and overweight/obese men (time×treatment, *P*=0.550; Fig. 4). As with cortisol and salivary alpha amylase, this lack of significant difference in the HR response between the groups was also indicated by the absence of a significant difference between the groups with regard to peak height, reactivity and AUC of the HR response (Table 4). The mean time to recovery did not differ significantly between the groups (Table 4). There was also no significant treatment effect, indicating that there was no significant overall difference between the groups (*P*=0.838).

There were no significant associations (data not shown) between measures of HR (pretreatment HR, peak HR, HR reactivity, AUC) and measures of adiposity (BMI, percentage body fat, waist circumference and waist-to-hip ratio).

## Discussion

The hypothesis that overweight/obese men will have a greater salivary cortisol, salivary alpha amylase and HR response to the TSST compared with age matched lean men was not supported. While there was a substantial increase in salivary cortisol, salivary alpha amylase and HR during the stress, this increase did not differ between the two groups. These findings show that, for men with a moderate level of obesity (BMI=30.6±0.6 kg/m^2^) who were otherwise healthy (men with severe illnesses were excluded from the study), the response of salivary cortisol, salivary alpha amylase and HR to a potent acute psychological stress was not compromised compared with that in lean men (BMI=23.5±0.3 kg/m^2^). Nevertheless, it is possible that differences between the groups may have been seen if there had been a higher degree of difference between the groups in the level of adiposity. Furthermore, there may be other stress-sensitive factors that are involved in the development of chronic disease, such as cytokines and/or opioids. Although these factors may have been influenced differently by the stressor in the two different groups, they were not measured in the current study. As such, it remains to be determined if these other factors may respond differently to stress.

The magnitude of the cortisol response that was found in the current experiment was substantial compared with the response in cortisol concentrations reported in past experiments. When both groups were combined, there was a 372% increase in cortisol in the current experiment. In humans, Therrien *et al*. (26) reported an 82% increase in cortisol after subjecting middle-aged men and women with different body weight and body fat profiles to the TSST. Benson *et al*. (22) reported an 83% increase in cortisol level in premenopausal obese and lean women after exposure to the TSST (22). The magnitude of the cortisol response observed in the current experiment also surpassed the increases in cortisol levels observed in experiments where exogenous agents have been used to stimulate the stress pathways. Ljung *et al*. (30) reported increases of around 200% in cortisol concentrations following treatment with CRH, and Pasquali *et al*. (31) reported increases in cortisol concentrations of 325% after CRH and AVP stimulation of age-matched premenopausal eumenorrheic women with different obesity phenotypes. Nevertheless, it is possible that more subtle differences in HPA axis activity would become evident with a less stressful task. Salivary alpha amylase also had a substantial reactivity (123%) in this experiment compared with earlier human studies that used salivary alpha amylase as a marker of the SAM system activity. For instance, Nater *et al*. (32) found a 50% increase in salivary alpha amylase levels when 24 healthy adults were exposed to the TSST. Increases in HR were also greater in the current experiment compared with some previous work. The HR response to stress in the current experiment (22%) is within the range of previously reported significant HR responses to TSST (30, 33, 34). These comparisons with other studies indicate that the response elicited by our TSST protocol was substantial and robust and may also indicate that the long resting period before stress in the current study had allowed these stress parameters to reach low resting levels.

The results also suggest that moderate levels of adiposity as observed in the current experiment may not compromise the response to acute psychological stress. We have shown in sheep that a large (23%) difference in the levels of adiposity (31.7±3.4% body fat vs 8.9±0.6%) is capable of eliciting a difference in stress ACTH, cortisol and epinephrine reactivity to psychological stress (35). By comparison, the differences in percentage body fat in the groups in the current study were more modest at 8% (lean=20.2±1.1% body fat vs overweight/obese=28.1±0.9%). Benson *et al*. (22) also reported a significantly enhanced cortisol response to psychological stress in severely obese (38.2±1.5 kg/m^2^) premenopausal women compared with their leaner (23.1±0.6 kg/m^2^) counterparts. BMI in the current study was 23.5±0.3 kg/m^2^ for lean and 30.6±0.6 kg/m^2^ for overweight/obese men. Similar to our findings, Ljung *et al*. (30) did not find a difference in HPA axis responses to psychological stress in individuals who had a BMI between 25 and 33 kg/m^2^. Furthermore, Therrien *et al*. (26) also did not find a difference in HPA axis responses between individuals (both men and women) who had a BMI of <27 kg/m^2^ and between 30 and 35 kg/m^2^. Thus, it appears that there may need to be a bigger difference in BMI or percentage body fat to observe a clear distinction in stress responsiveness between lean and obese individuals. Now that a significant proportion of the current population is either overweight or obese, the study groups of this current experiment may be particularly clinically relevant. Nevertheless, this experiment provides no evidence that salivary cortisol, salivary alpha amylase and HR responsiveness to psychological stress are influenced by this level of adiposity. Further research might need to compare morbidly obese individuals with lean individuals to further characterise differences in stress responsiveness.

It is also possible that factors other than those associated with the SAM system and the HPA axis are important in determining health risks of responses to psychological stress. For instance, inflammatory cytokine responses (1) and opioid pathways may play a role in mediating the stress response (14). Therefore, it is possible that various factors that were not investigated in this experiment are involved in mediating the effects of stress in lean and overweight/obese men. Further research is required to consider a potential role for these other mediators.

A common criticism of stress experiments is the lack of potency of the stressor (28, 36). The stressor implemented in this experiment was able to elicit substantial responses from both the HPA axis and the SAM system. Another strength of this study was the frequent sampling during the period of stress, which enabled detailed profiling of how the stress parameters responded during the stressor. The lengthy rest time before the administration of the stressor was another benefit given that this reduces the likelihood of the stress systems being already activated at the commencement of the sampling period. Furthermore, age is known to influence stress responsiveness (37). The participants in the current experiment were middle aged and older. Therefore, younger age groups could also be considered.

## Figures and Tables

**Table 1:** Mean (±s.e.m.) baseline descriptive characteristics and food intake in lean and overweight/obese men.

	**Lean** (*n*=19)	**Overweight/obese** (*n*=17)	***P* value** ^a^
Age (years)	63.3±1.1	61.1±1.1	0.166
Height (cm)	172.0±1.4	174.8±1.4	0.179
Weight (kg)	69.7±1.6	93.8±2.3	<0.001
BMI (kg/m^2^)	23.5±0.3	30.6±0.6	<0.001
BMI range	20.5–24.9	27.2–34.3	
% Fat	20.2±1.1	28.1±0.9	<0.001
% Lean	79.3±1.1	72.0±0.9	<0.001
Waist circumference (cm)	86.1±1.5	106.9±1.5	<0.001
Hip circumference (cm)	97.5±1.2	109.2±1.3	<0.001
WHR	0.88±0.01	0.98±0.01	<0.001
SBP (mmHg)	119.1±3.3	129.0±2.8	0.030
DBP (mmHg)	67.7±2.1	74.5±2.0	0.027
MAP (mmHg)	85.3±2.4	93.3±2.2	0.022
HR (bpm)	64.2±3.0	64.4±2.6	0.968
Total energy (kJ)	2895±245	3015±235	0.728
Protein (g)	27.2±2.1	29.9±2.5	0.396
Carbohydrate (g)	65.2±6.4	73.0±5.4	0.362
Fat (g)	37.2±4.3	35.7±4.3	0.801
Sodium (mg)	1080±83	1260±118	0.215

% Fat, percentage body fat; % Lean, percentage lean mass; WHR, waist-to-hip ratio; SBP, systolic blood pressure; DBP, diastolic blood pressure; MAP, mean arterial pressure; HR, heart rate.

a Univariate ANOVA.

**Table 2:** Mean (±s.e.m.) pretreatment cortisol, peak height of cortisol, cortisol reactivity, AUC and recovery time for lean and overweight/obese men.

	**Lean** (*n*=19)	**Overweight/obese** (*n*=17)	***P* value** ^a^
Pretreatment (μg/dl)	0.29±0.02	0.28±0.02	0.788
Peak height (μg/dl)	1.52±0.22	1.21±0.15	0.254
Reactivity (μg/dl)	1.23±0.21	0.93±0.15	0.263
AUC (μg/dl per min)	55.3±10.3	38.7±7.7	0.118
Recovery time (min)	103.3±6.0	101.8±5.9	0.867

AUC, area under the curve.

a Univariate ANOVA.

**Table 3:** Mean (±s.e.m.) pretreatment salivary alpha amylase, peak height, salivary alpha amylase reactivity, AUC and recovery time for lean and overweight/obese men.

	**Lean** (*n*=19)	**Overweight/obese** (*n*=17)	***P* value** ^a^
Pretreatment (U/ml)	112.1±16.1	140.8±16.5	0.224
Peak height (U/ml)	267.3±55.5	295.6±41.2	0.690
Reactivity (U/ml)	155.1±51.2	154.9±31.6	0.997
AUC (U/ml per min)	5221±2735	3131±1525	0.523
Recovery time (min)	74.1±7.7	60.6±9.2	0.267

AUC, area under the curve.

a Univariate ANOVA.

**Table 4:** Mean (±s.e.m.) pretreatment HR, peak height of HR, HR reactivity, AUC and recovery time for lean and overweight/obese men.

	**Lean** (*n*=17)	**Overweight/obese** (*n*=15)	***P* value** ^a^
Pretreatment (bpm)	64.4±2.2	64.6±3.5	0.964
Peak height (bpm)	80.2±4.1	76.9±4.0	0.570
Reactivity (bpm)	15.8±2.5	12.3±1.7	0.270
AUC (beats/min^2^)	148.1±113.6	31.3±71.6	0.406
Recovery time (min)	40.1±3.7	45.5±5.9	0.440

AUC, area under the curve.

a Univariate ANOVA.
